# Patient-specific digital twins in aortic disease: integrating computational hemodynamics, immune profiling, and precision endovascular strategy

**DOI:** 10.3389/fcvm.2026.1812347

**Published:** 2026-05-11

**Authors:** Rasit Dinc, Nurittin Ardic

**Affiliations:** 1 INVAMED Medical Innovation Institute, New York, NY, United States; 2 Med-International UK Health Agency Ltd., Leicestershire, United Kingdom

**Keywords:** aortic aneurysm, artificial intelligence, biomechanics, computational hemodynamics, digital twin, endovascular strategy, inflammation, risk stratification

## Abstract

Aortic aneurysms and acute aortic syndromes continue to be significant contributors to cardiovascular morbidity and mortality; however, current risk stratification and treatment timing rely heavily on static anatomical thresholds that do not fully reflect the dynamic biology and mechanics of disease progression. Patient-specific digital twins offer a unifying paradigm where multimodal patient data are integrated into a continuously updated computational representation of an individual aorta to predict trajectories and support precise decision-making. In this review, we propose a five-domain architecture for an aortic digital twin (1): a structural-biomechanical substrate that reconstructs patient geometry and predicts wall stress and remodeling tendency; (2) computational hemodynamics to quantify flow-derived descriptors such as wall shear stress, oscillatory shear, and stagnation; (3) immunobiological integration to incorporate inflammatory activity, immune cell heterogeneity, and proteolytic remodeling signals; (4) predictive intelligence that combines multimodal features, generates individualized growth and complication predictions through uncertainty quantification, and updates predictions with longitudinal data; and (5) a sophisticated endovascular strategy layer that translates twin outputs into procedural planning, device selection, and risk-corrected surveillance. This framework highlights how the bidirectional link between inflammation and mechanics can be functionalized as a feedback ecosystem rather than a set of independent analyses, and outlines the evidence requirements for clinical translation, including validation pathways, workflow integration, and management considerations. By shifting the clinical focus from “Is the aorta large enough?” to “How will this patient's disease develop, and how can we best modify the course?”, aortic digital twins can enable earlier, more individualized, and more lasting prevention of adverse aortic events.

## Graphical Abstract

**Figure:**
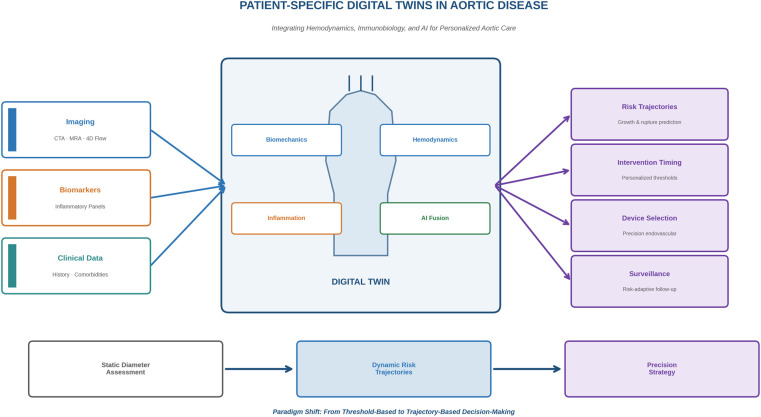
Schematic overview of a patient-specific digital twin framework in aortic disease, integrating imaging, hemodynamics, immunobiology, and artificial intelligence to generate individualized risk trajectories and guide precision endovascular decision-making.

## 1 Introduction

Aortic aneurysms and acute aortic syndromes remain major causes of cardiovascular morbidity and mortality, despite significant advances in imaging, perioperative care, and endovascular technologies (1). Current clinical decision-making relies heavily on population-derived anatomical thresholds, most notably maximum aortic diameter, and intermittent imaging follow-up. While practical and guideline-compliant, this approach does not fully reflect the individualized biology and biomechanics of aortic wall destabilization and contributes to persistent uncertainty in patient-specific intervention timing, follow-up intensity, and procedural strategy (1–3).

Over the past two decades, computational and data-driven methods have expanded the mechanistic perspective beyond geometry. Patient-specific computational hemodynamics has enabled the mapping of wall shear stress (WSS), oscillatory shear, flow complexity, and recirculation patterns that may be associated with endothelial activation, thrombus evolution, and regional remodeling (4, 5). In parallel, biomechanical modeling has aimed to predict peak wall stress and stress concentrations, which can differ significantly from anchor-based assessments, reflecting heterogeneity in wall properties, thrombus load, and geometry (6, 7). Immune-mediated extracellular matrix disruption, driven by inflammation and leukocyte trafficking, protease activity, and cytokine signaling, has emerged as key determinants of aneurysm growth and instability, motivating the search for biomarker and molecular imaging correlations of “biological risk” (8, 9).

Despite these advances, most modeling approaches remain operationally independent. Computational fluid dynamics provides high-resolution hemodynamic maps, often without incorporating patient-specific inflammatory status or rates of biological remodeling. Biomarker and molecular imaging studies quantify systemic or regional inflammation without full mechanistic context. Meanwhile, machine learning models can classify risks or predict outcomes from large datasets, but often reflect correlation-driven predictors with limited mechanistic linkage and variable generalizability across protocols and populations (10, 11). In practice, the aortic disease phenotype evolves over time, but many models operate as single-timepoint snapshots.

Digital twin technology refers to a continuously updated, patient-specific computational representation that integrates real-world data for prediction and decision support. It offers a unifying conceptual framework to address this fragmentation. At present, major limitations of clinical digital twin implementations include incomplete multimodal data availability, variability in acquisition protocols, uncertainty in model assumptions, computational burden, and the need for prospective validation in real-world workflows. In engineering, this technology is a dynamic computational copy of a physical system updated with real-world data to support prediction, optimization, and decision-making processes. In cardiovascular medicine, recent reviews have highlighted the importance of digital twins with the potential to generate continuously updated, patient-specific predictions that can inform clinical actions by integrating mechanistic modeling with multimodal clinical data streams (imaging, physiology, laboratory markers, and outcomes) (12–14). Importantly, the early cardiovascular twin literature emphasizes that clinical translation relies more on integration, validation, uncertainty communication, and workflow applicability than on isolated model complexity (12, 14).

In aortic disease, emerging studies demonstrate the feasibility of high-accuracy patient-specific digital twins for the thoracic aorta, including fluid-structure interaction approaches aimed at simulating intervention scenarios and device-aorta interactions (15). However, most “aortic twin” applications remain dominated by geometry and hemodynamics, with limited integration of immunobiological factors that directly influence wall weakening, remodeling kinetics, and post-intervention behavior. This gap has significant consequences: mechanics and biology operate as an interconnected system; hemodynamic forces regulate endothelial activation and leukocyte recruitment; inflammatory proteolysis alters wall material properties and failure risk; structural remodeling reshapes flow fields; and endovascular devices further disrupt both mechanical and biological domains.

Therefore, we propose that a clinically meaningful, patient-specific aortic digital twin should go beyond static simulation or independent prediction, with the following characteristics: (i) multimodal, integrating imaging-derived anatomy with computational hemodynamic, biomechanical, and immunoprofiling; (ii) dynamic, updated with serial imaging, biomarkers, and clinical events; (iii) mechanistically informed, incorporating biological and physical factors rather than relying solely on statistical correlation; and (iv) actionable, capable of guiding surveillance intensity, repair timing, and precision endovascular strategy (12, 14). In this paradigm, the clinical question shifts from whether an aorta exceeds a fixed threshold to how an individual aorta evolves under its unique mechanical loads and inflammatory status, and how alternative interventions might alter this trajectory.

The following sections outline a multilayered architecture for patient-specific digital twins in aortic disease, highlighting the integration of structural-biomechanical substrate, computational hemodynamics, immunobiological profiling, and adaptive predictive intelligence into a continuous feedback ecosystem to support precise endovascular care.

To improve readability and emphasize clinical applicability, Table 1 summarizes the proposed aortic digital twin architecture across Domains 1–5, mapping representative inputs, core computational processes, and key outputs to the clinical decisions they aim to inform. This table presents a high-level implementation plan highlighting how structural-biomechanical modeling, computational hemodynamics, immunobiological integration, predictive intelligence, and virtual intervention planning are interconnected within a longitudinally updated decision support workflow.

**Table 1:** Multimodal input parameters and functional components of a patient-specific aortic digital twin.

Domain	Primary inputs	Computational processes	Key outputs	Clinical decision support
1. Structural-biomechanics	CTA/MRA geometry; ILT segmentation; wall thickness estimates	Finite element analysis; growth and remodeling models; material feature extraction	Peak wall stress maps; stress concentration regions; expansion trajectory estimates	Rupture risk stratification; procedure timing support
2. Computational hemodynamics	Patient anatomy; boundary conditions; 4D Flow MRI (optional)	CFD simulation; WSS/OSI calculation; flow complexity metrics; uncertainty quantification	Hemodynamic field maps; shear stress distributions; recirculation/stagnation regions	Identification of high-risk flow environments; support for hemodynamic optimization strategies (e.g., blood pressure control)
3. Immunobiological integration	Circulating biomarkers; cytokine/MMP panels; monocyte subgroups; ^1^⁸F-FDG PET (optional)	Systemic immune profiling; spatial inflammation mapping; biology-mechanics matching; post-intervention inflammatory dynamics	Inflammatory activity estimates; proteolytic activity indices; remodeling susceptibility modulators	Biomarker-informed monitoring; support for adjuvant medical strategy evaluation; post-EVAR biological monitoring
4. Predictive intelligence	Multi-domain features; serial imaging; longitudinal biomarkers; clinical covariates	Multimodal data fusion; trajectory prediction; Bayesian update; explainability methods	Growth predictions with uncertainty; predicted trajectory milestones (e.g., threshold timing); driver attribution (risk decomposition)	Personalized monitoring intervals; intervention decision support; transparent risk communication
5. Precision endovascular strategy	Digital twin status; instrument characteristics; landing zone geometry; estimated hemodynamics	Virtual device deployment; seal-zone assessment; flow redistribution simulation; endoleak/device-related complication prediction	Device sizing/selection recommendations; endoleak risk predictions; sac regression forecasts	Procedure planning; device selection support; risk-adaptive postoperative surveillance

## 2 Multilayer architecture of a patient-specific aortic digital twin

**Figure 1:**
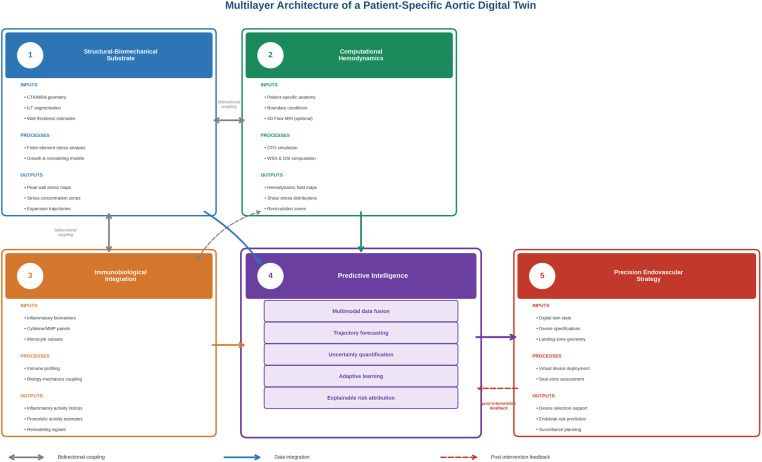
Multilayer architecture of patient-specific aortic digital twin. The framework integrates five interconnected domains: (1) structural-biomechanical substrate, (2) computational hemodynamics, (3) immunobiological integration, (4) predictive intelligence, and (5) precision endovascular strategy. Domains 1–3 generate mechanistic representations of geometry, wall stress, flow patterns, and inflammatory activity. Domain 4 performs multimodal data fusion, trajectory prediction, uncertainty quantification, and adaptive learning to generate individualized risk projections. Domain 5 translates these outputs into intervention planning, device selection, and surveillance optimization. Arrows indicate data integration and bidirectional coupling between domains.

The proposed digital twin framework is organized as a hierarchical and interdependent five-domain architecture designed to transform static anatomical representations into dynamic, trajectory-based clinical intelligence. Rather than functioning as isolated analytical modules, these domains operate as a unified ecosystem where structural mechanics, computational hemodynamics, and immunobiological activity continuously inform predictive modeling and procedural strategy. This architecture enables iterative data integration, bidirectional feedback, and incremental model refinement throughout the patient's disease course. Figure 1 illustrates the conceptual architecture and directional data flow between these domains.

### 2.1 Domain 1: structural-biomechanical substrate

A patient-specific aortic digital twin begins with an accurate representation of geometry, composition, and material behavior, as these structural features govern local stress distributions, remodeling trajectories, and (downstream) the response to hemodynamic and inflammatory stimuli. In practice, this Domain comprises three interconnected elements: (i) patient-specific geometry and tissue segmentation, (ii) biomechanical characterization and failure-related metrics, and (iii) growth and remodeling (G&R) formulations that allow the substrate to evolve over time rather than remaining a static snapshot.

#### 2.1.1 Patient-specific geometry and tissue segmentation

High-quality anatomical reconstruction is typically obtained from CTA or MRA, and segmentation extends beyond luminal boundaries to include intraluminal thrombus (ILT), wall contours where possible, branch vessel orifices, and calcification load. Since digital twins are designed for serial updating, reproducible segmentation workflows are crucial for longitudinal consistency, especially where “small” geometric changes can significantly alter calculated wall stress fields and subsequent predictions. ILT also creates a dynamic thrombo-inflammatory niche characterized by neutrophil extracellular trap (NET) formation, proteolytic enzyme release, and localized oxidative stress, all of which can enhance matrix disruption and wall weakening (16). CTA generally provides higher spatial resolution and superior delineation of the wall and intraluminal thrombus compared with MRA and is therefore more commonly used for high-fidelity geometric reconstruction. Importantly, reconstruction fidelity depends not only on spatial resolution but also on segmentation accuracy and inter-scan consistency. While CTA is typically preferred for detailed anatomical modeling, MRA offers advantages for longitudinal tracking and flow-sensitive assessment. In this context, reproducibility between serial images may be more critical than adhering to fixed spatial resolution thresholds.

Deep learning methods are increasingly being used to automate the segmentation of AAA sections (e.g., lumen, ILT, outer wall), addressing the scalability limitations of manual delimitation and improving suitability for clinical practice (17). For ILT in particular, recent reviews highlight rapid progress in DL-based ILT segmentation from CTA, emphasizing generalizability across scanners and protocols and the importance of standardized assessment criteria for clinical translation (18). These advances are particularly important for digital twins because ILT is not merely a “geometric add-on”: it modifies effective wall stress and is closely linked to local biology, including hypoxia, proteolysis, and inflammatory cell recruitment; these features influence remodeling rates and wall attenuation.

##### 2.1.1.1 Practical implication for the twin

Geometry is not a one-time preprocessing step. It is a representation of the patient's evolving state and must be updated with each new scan. This enables recalibration of stress distributions and provides a stable framework upon which hemodynamic and immunobiological layers can be mapped.

#### 2.1.2 Biomechanical characterization and rupture-related measures

Biomechanical analysis aims to predict stress and strain distributions in patient-specific aortic walls and obtain rupture-related measures that yield better results than diameter-based measurements alone. Basic studies have shown that peak wall stress in AAA cohorts can discriminate rupture risk better than maximum diameter (6), and subsequent syntheses have evaluated how model complexity affects predictability and clinical utility (7, 19). Therefore, a current patient-specific twin model should treat biomechanics not as a purely deterministic simulation, but as a calibrated inference problem: inputs such as wall thickness, ILT distribution, calcification, and boundary loading should be explicitly represented, and outputs should be reported with ambiguity where assumptions are unavoidable. This emphasis is consistent with a broader shift toward clinically usable biomechanical risk assessment tools, including pipelines that predict rupture indices by relating calculated wall stress to statistical indicators of wall strength (20).

##### 2.1.2.1 Practical implication for twin

Biomechanical outputs should be stored as timestamped fields (e.g., peak wall stress, stress concentrations in bending zones, regional strain indicators) so that they can be longitudinally compared and used as features in the predictive intelligence domain.

#### 2.1.3 Growth and remodeling as an evolving substrate

A defining characteristic of digital twins is their co-evolution with the patient. For aortic disease, evolution reflects the combined effects of hemodynamics, inflammation, and mechanobiology on matrix transformation, cellular activity, and wall material properties. Constrained mixing theory and related mechanobiological frameworks provide a principled way to represent growth and remodeling (G&R) as a result of component-level production/degradation under mechanical stimuli (21, 22).

Within the digital twin architecture, G&R modeling is not primarily concerned with perfect mechanistic accuracy in the first iteration; rather, it provides a conceptual and computational interface for integrating biological signals into structural evolution. For example, immune-derived indicators of proteolytic activity or inflammation load can be mapped to remodeling rate parameters, while serial imaging updates recalibrate the geometry and provide exogenous constraints on predicted expansion trajectories. In this way, the structural substrate becomes a living component of the twin model, capable of capturing patient-specific trajectories rather than relying solely on population averages.

##### 2.1.3.1 Practical implication for the twin model

Explicitly representing structural evolution enables clinically meaningful questions such as trajectory prediction (growth acceleration vs. stability), intervention timing, and subsequent virtual intervention testing where device-induced mechanical changes can be translated into remodeling predictions.

### 2.2 Domain 2: computational hemodynamics

Computational hemodynamics is the dynamic engine of the aortic digital twin: it translates patient-specific anatomy and boundary conditions into spatially resolved descriptors of flow, near-wall forces, and transport events that may be linked to endothelial activation, thrombus biology, and regional remodeling. In the context of the digital twin, the aim is not only high-accuracy simulation but also hemodynamic inference that is repeatable, updatable, and ambiguity-aware, capable of being re-run as anatomy and physiology evolve longitudinally (5).

#### 2.2.1 Patient-specific flow simulation: inputs, boundary conditions, and model selection

Aortic computational fluid dynamics (CFD) pipelines typically start from geometry derived from CTA/MRA (Domain 1) and require careful determination of input and output boundary conditions to achieve clinically meaningful results. Reviews focusing on aortic aneurysm and dissection modeling highlight that boundary condition selections (e.g., patient-specific input waveform, output impedances/Windkessel models, pressure calibration) can determine the predicted wall shear metrics and flow patterns, especially in complex geometries and dissection lumens (5, 23). Clinically, boundary conditions can be derived from patient-specific measurements when available, such as Doppler echocardiography, phase-contrast MRI, or catheter-based pressure recordings. In their absence, population-based waveform templates scaled according to patient-specific physiological parameters (e.g., heart rate, blood pressure, vessel diameter) are commonly used.

For digital twins, personalization of input waveforms and physiological loading is particularly important, as the same anatomy can generate significantly different shear environments under different cardiac outputs, blood pressures, or flow splits. Recent methodological studies suggest practical strategies for scaling input flow waveforms to improve patient specificity in cases where direct measurements are unavailable or incomplete (24).

A crucial practical decision is to select the level of accuracy required for a given clinical question. While laminar pulsatile CFD is sufficient for many aneurysm applications, impaired flow, transitional features, and complex secondary flow structures can arise in dilated segments, sharp curvatures, or dissection channels. Accordingly, modern aortic CFD studies increasingly advocate aligning model complexity with the target endpoint (e.g., virtual intervention assessment with hemodynamic descriptors for growth prediction) and transparently reporting model assumptions (5, 23).

#### 2.2.2 Image-based hemodynamics: 4D flow MRI for personalization and validation

A distinctive advantage in the aorta is the increasing maturity of 4D flow MRI, which provides time-resolved velocity fields and enables direct estimation of hemodynamic parameters, facilitating both boundary condition personalization and model validation. Current reviews of thoracic aortic 4D flow MR summarize clinical and research applications, including the characterization of anomalous flow jets, helicity, and regional shear patterns in dilation and aneurysm phenotypes (25).

Importantly, 4D flow-derived WSS is increasingly being evaluated as a marker associated with aortic remodeling and growth, and recent syntheses reveal that, while not methodologically trivial, WSS estimation can provide biologically relevant spatial signals for risk stratification (26). In addition, observational studies have begun to correlate 4D flow hemodynamic features in ascending aortic dilation with circulating biomarkers, supporting the broader “bio-hemodynamic coupling” proposition that aortic flow environments may be associated with systemic molecular signatures (27). However, WSS estimates obtained from 4-dimensional flow MRI should be interpreted with caution, as spatial resolution and noise limitations can affect accuracy, and validation against high-resolution CFD remains an ongoing area of research.

##### 2.2.2.1 Digital twin implication

Image-based hemodynamics, especially when serial imaging allows for recalibration of both anatomical and physiological loading, helps transform CFD from a one-off academic simulation into a clinically reliable, updatable module when embedded into a longitudinal update cycle.

#### 2.2.3 Hemodynamic biomarkers: what the twin should compute and store

For aneurysm and dissection phenotypes, the digital twin should compute hemodynamic descriptors that are (i) repeatable between updates, (ii) physiologically interpretable, and (iii) reasonably linked to mechanobiology. These typically include WSS magnitude and vectors, OSI, flow reversal/residence time proxies, and flow complexity indices. Recent aorta-focused CFD reviews are providing consensus on these outputs and summarizing the relationships that emerge with progression endpoints (5, 23).

For dissection, hemodynamic descriptors may also include true/false lumen flow splitting, entry rupture jet characteristics, and pressure/energy loss proxies. Since dissection growth and remodeling are sensitive to entry profile representation, patient-specific entry flow characterization can significantly influence the predicted expansion patterns; Modern studies in TBAD modeling highlight the value of detailed input velocity profiles and patient-specific conditions (28).

##### 2.2.3.1 Digital twin implication

Hemodynamic outputs should be treated as timestamped fields (not single summary numbers) so that comparison between surveillance scans and integration as features in the predictive intelligence domain can be achieved.

Domains 1–3 constitute the biological computational infrastructure of the patient-specific aortic digital twin: structural geometry and mechanics provide the evolving physical skeleton; hemodynamics characterize the dynamic flow forces acting on this infrastructure; and immunobiological profiling quantifies the biological factors of wall degradation and remodeling. Critically, these Domains do not operate independently; they form a continuous feedback ecosystem where hemodynamics influences inflammation, inflammation modulates mechanics, and structural evolution reshapes flow fields. Domain 4 introduces the predictive intelligence layer that learns from this multimodal ecosystem to generate patient-specific risk trajectories and guide clinical decision-making processes.

#### 2.2.4 Uncertainty quantification and sensitivity analysis: making CFD clinically trustworthy

A clinical digital twin should convey not only predictions but also confidence. Hemodynamic metrics, especially WSS and derived indices, are sensitive to segmentation, boundary conditions, blood rheology assumptions, and numerical adjustments. Uncertainty quantification (UQ; estimation of prediction confidence and variability due to model assumptions and input uncertainty) frameworks and sensitivity analyses provide a principled way to identify which inputs dominate output variability and report confidence intervals instead of single deterministic values. A cardiovascular modeling study published in Frontiers demonstrates how UQ can be integrated into coupled CFD simulations for hemodynamic outputs and provides a template for how uncertainty reporting can be operationalized (29, 30).

##### 2.2.4.1 Digital twin implication

UQ should be a first-class element in the hemodynamic module, especially when hemodynamic biomarkers are used downstream for risk estimation, immunomechanical matching, or intervention planning.

### 2.3 Domain 3: immunobiological integration: the biological driver of structural instability

While the structural-biomechanical substrate describes the mechanical state of the aorta and computational hemodynamics characterize local flow forces, immunobiological activity governs the rate and pattern of wall degeneration. A patient-specific aortic digital twin excluding inflammation captures the geometry and mechanics but risks missing key biological accelerators of aneurysm expansion, focal attenuation, and post-intervention remodeling failure. Aortic aneurysm progression is increasingly recognized as a chronic inflammatory process involving leukocyte recruitment, macrophage polarization, extracellular matrix disruption, and dysregulated repair (8, 9, 31). Contemporary immunobiology further emphasizes that the inflammatory load is not uniform (32). Immune cell heterogeneity, particularly within monocyte and macrophage subgroups, plays a central role in aneurysm progression, extracellular matrix disruption, and wall destabilization (33).

In particular, proteolytic activity via matrix metalloproteinases such as MMP-2 and MMP-9 contributes to elastin degradation and collagen remodeling, altering wall tensile properties and biomechanical behavior (9).

These biological processes are regionally heterogeneous and may be compatible with local hemodynamic and thrombus environments; this strengthens the rationale for explicitly integrating immune signals into the digital twin architecture rather than treating them as extrinsic correlations. Despite its conceptual importance, immunobiological integration currently faces practical limitations. Serial high-resolution immunoprofiling and molecular imaging (e.g., ^1^⁸F-FDG PET) are not routinely available in most clinical workflows, and standardized protocols for longitudinal integration are still under development. These constraints highlight the need for scalable surrogate biomarkers and modular implementation strategies.

#### 2.3.1 Systemic immune profiling: circulating biomarkers as indicators of biological Status

Circulating biomarkers provide a practical representation of systemic inflammatory and proteolytic activity. Reported associations include inflammatory cytokines (e.g., IL-6, TNF-α), chemokines (e.g., MCP-1), and matrix transformation markers; this is consistent with a model where sustained low-grade inflammation contributes to expansion and instability (8, 31).

From a digital twin perspective, the key point is not that any single biomarker is definitive, but that immune markers can serve as dynamic inputs that contextualize mechanistic risk. Specifically, consistently high inflammatory profiles likely correspond to higher rates of effective remodeling, while refinement of biomarker trajectories can support lower risk predictions and longer surveillance intervals when structural and hemodynamic conditions are otherwise stable.

Because immune activity fluctuates over time due to medical treatment, comorbidities, and disease evolution, digital twinning should prioritize serial profiling over isolated measurements. Longitudinal biomarker integration allows the twin to differentiate between stable low-level inflammation and ascending immune activation that may herald accelerated wall weakening by converting static values into patient-specific trajectories. This temporal biological signal becomes particularly valuable when integrated with serial imaging, as it can help explain why two patients with similar diameters might differ in growth rates and clinical risk.

##### 2.3.1.1 Digital twin implication

Circulating immune profiles should be considered not merely as independent predictors, but as time-stamped covariates influencing uncertainty-sensitive predictions (Domain 4) and parameterization of structural evolution (Domain 1).

#### 2.3.2 Molecular imaging of inflammation: spatialization of biology

While blood biomarkers provide systemic context, molecular imaging allows for partial spatial localization of inflammation. ^18^F-FDG PET/CT has been investigated as a marker of metabolic activity and inflammation in aneurysm tissue, and observational relationships between increased uptake and aneurysm expansion have been identified in the translational literature (8).

In digital twinning, the key advantage of molecular imaging is not only to add another data point but also to enable the mapping of inflammatory “hot spots” to structural and hemodynamic domains.

In particular, signals from PET can be mapped to the reconstructed aorta and interpreted together with hemodynamic descriptors (e.g., low/oscillatory slip regions) and biomechanical outputs (e.g., stress concentrations) obtained from CFD. This allows the digital twin to move beyond a purely global “high inflammation” label and towards a spatially explicit interpretation where local biology, local mechanics, and local flow disruption can be assessed together.

##### 2.3.2.1 Digital twin implication

Inflammation imaging (where available) should be considered not as a purely global risk marker, but as a spatial layer that can be recorded into the geometry and used to regulate region-specific remodeling assumptions.

#### 2.3.3 Biology-mechanics coupling: a bidirectional system

The fundamental conceptual contribution of immunobiological integration is the recognition of a bidirectional coupling between mechanical forces and biological activity. Impaired hemodynamics can promote endothelial dysfunction, leukocyte adhesion, and inflammatory signaling; inflammatory infiltration then weakens structural integrity by increasing proteolytic activity and extracellular matrix degradation; altered material properties reshape wall stress distributions; and evolving geometry and wall properties then alter flow patterns. In other words, hemodynamics influences biology, and biology alters mechanics; creating a feedback loop rather than a unidirectional pathway (8, 9).

**Figure 2:**
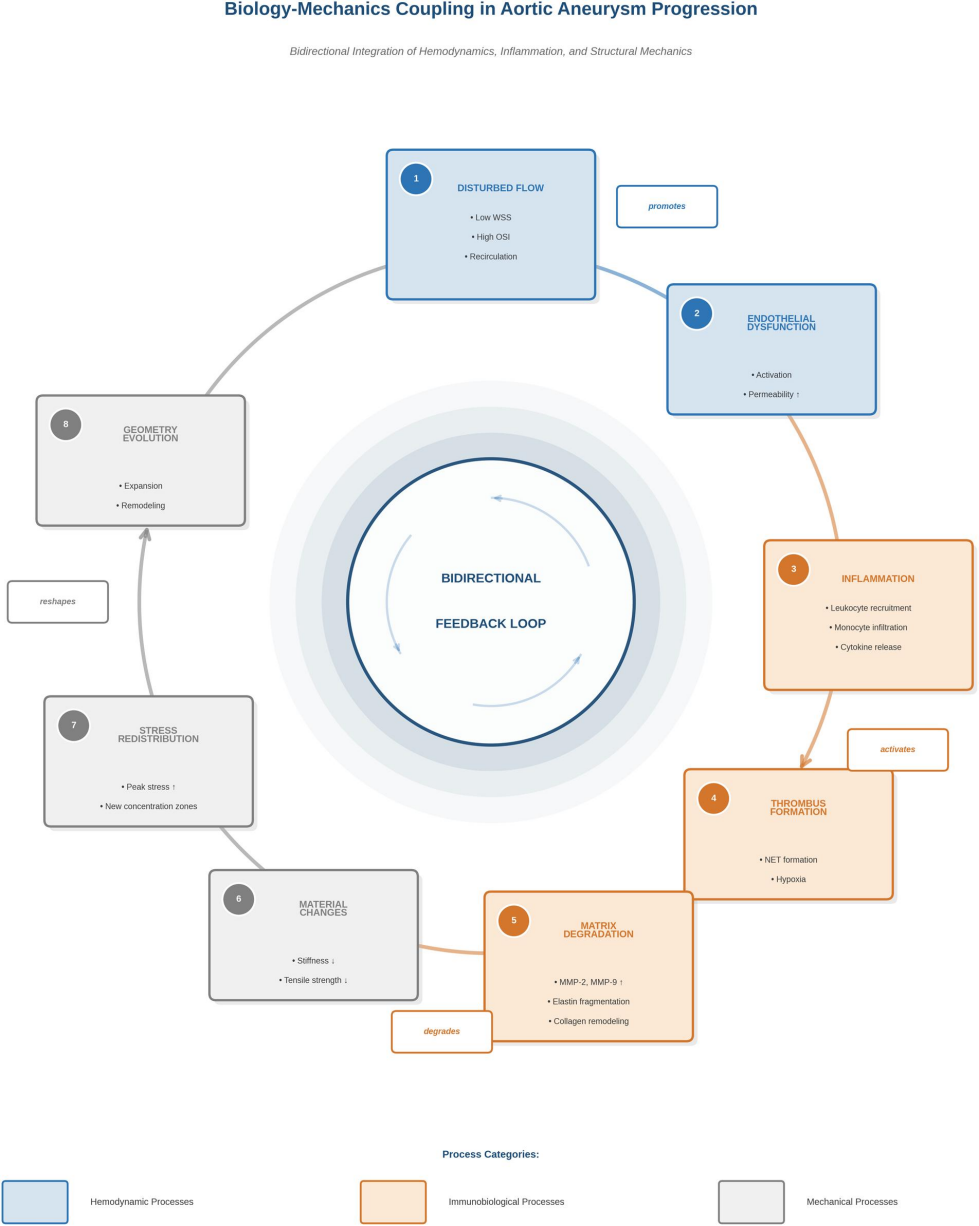
Biology-mechanics coupling in aortic aneurysm progression. Impaired flow patterns, characterized by low wall shear stress and high oscillatory shear index, promote endothelial dysfunction and immune activation. Inflammatory processes, including leukocyte recruitment and proteolytic matrix degradation, alter material properties and redistribute wall stress. These mechanistic changes reshape the aortic geometry, further altering local hemodynamics and maintaining a bidirectional feedback loop. Hemodynamic (blue), immunobiological (orange), and mechanics (gray) processes are shown to highlight the interdomain coupling.

The bidirectional interaction between hemodynamics, inflammation, and structural remodeling can be conceptualized as a continuous feedback loop rather than a linear cascade. This system-level linkage is shown in Figure 2.

This coupled view becomes even more important when considering thrombosis. Intraluminal thrombus can function as a thrombo-inflammatory microenvironment, and NET-associated pathways provide a mechanistic bridge between thrombosis, inflammation, and tissue damage (16).

Therefore, regions where disrupted flow coexists with thrombus interfaces and high inflammatory signals may represent biologically vulnerable zones that cannot be determined solely by diameter or mechanistic properties.

##### 2.3.3.1 Digital twin implication

Mapping rules that explicitly link hemodynamic environments, immune activity, and evolving material properties must be coded to support mechanistically informed trajectory prediction rather than correlation-based risk estimation.

#### 2.3.4 Post-intervention immune dynamics

Endovascular repair alters mechanics and flow, but does not automatically eliminate biological processes that promote degeneration. Device-wall interactions, altered flow patterns, and thrombus remodeling can disrupt inflammatory pathways, and patients may exhibit heterogeneous post-procedure biological responses despite similar technical outcomes. Therefore, immunobiological integration helps ensure that the twin does not equate mechanical exclusion with biological stabilization by default.

Serial immunoprofiling after EVAR/TEVAR can serve as a recalibration signal within the twin model: persistent inflammatory activation may indicate ongoing proteolytic activity and incomplete biological stabilization, while decreased inflammatory indices may be consistent with favorable remodeling and sac regression trajectories (8).

Importantly, the divergence between mechanical success (e.g., technically adequate sealing) and biological persistence may signal a risk of delayed complications and motivate closer monitoring or adjunctive medical strategies.

##### 2.3.4.1 Digital twin implication

Post-intervention immune trajectories should be used as feedback signals updating predictions and uncertainty, allowing the twin model to adapt to patient-specific biological responses rather than relying on population averages.

#### 2.3.5 Translational impact: from biomarkers to model parameters

The aim of Domain 3 is not to catalog biomarkers, but to functionalize biology within the evolving model state of the digital twin. Practically, this involves mapping composite immune activity derived from circulating profiles and, if present, inflammation imaging, to remodeling-related parameters and uncertainty limits used in structural evolution and trajectory prediction. When follow-up imaging shows faster or slower expansion than predicted, the twin can recalibrate the weight assigned to immune signals and adjust prediction ranges accordingly, in line with the adaptive learning strategy outlined in Domain 4. Embedding immunobiology into structural and hemodynamic modeling helps explain a fundamental clinical observation: two patients with similar aneurysm diameters can follow distinctly different courses depending on their inflammatory status and proteolytic activity (31, 32).

This biologically informed representation is essential for the shift from diameter-centered stratification to individualized, mechanistically based prediction.

##### 2.3.5.1 Digital twin implication

Immune traits should be encoded as dynamic modulators of growth/remodeling assumptions, rather than added as static correlations, and integrated into multimodal risk trajectories.

### 2.4 Domain 4: predictive intelligence and adaptive learning

If Domains 1–3 describe the evolving state of the aortic system [geometry and mechanics (Domain 1), flow forces (Domain 2), and immunobiological activity (Domain 3)], Domain 4 describes how this multimodal state is translated into interpretable predictions, updated longitudinally, and translated into clinically actionable guidance. Recent cardiovascular digital twin literature highlights those digital twins become clinically meaningful not simply by adding more modules, but by providing consistent integration, uncertainty-aware updating, and decision support aligned with real-world clinical workflows (12–14).

A key requirement is multimodal integration across scales. The predictive intelligence layer should integrate imaging-derived morphology (e.g., diameter, volume, ILT load and morphology), biomechanical domains (e.g., peak wall stress and stress gradients), hemodynamic descriptors (e.g., WSS distribution, OSI, flow complexity, and recirculation indicators), immunobiological signals (e.g., circulating inflammatory/proteolytic markers and inflammation imaging where available), and standard clinical covariates (age, sex, blood pressure, smoking, concomitant diseases, genetics). Rather than treating these inputs as independent predictors, the digital twin framework acknowledges interdomain interaction with the assumption that aneurysm progression is driven by conjugated biomechanical processes.

Hybrid modeling approaches are therefore particularly suitable: mechanistic simulations provide structured, physiologically interpretable features, while machine learning supports nonlinear fusion, temporal prediction, and adaptive updating. This positioning also addresses a common limitation of independent AI predictors: high apparent performance with limited mechanistic basis and variable generalizability across centers (10, 11). From a practical standpoint, hybrid modeling can take multiple forms. Mechanistic simulations (e.g., wall shear stress gradients or biomechanical stress fields derived from CFD) can be used as structured features for machine learning models that predict growth or complication risk. Conversely, machine learning approaches can aid in the calibration of boundary conditions, the estimation of missing parameters, or the acceleration of surrogate simulations of physics-based models. This bidirectional integration allows for combining mechanistic interpretability with data-driven adaptability.

In the digital twin, AI does not replace physics-based modeling; it is the integrative layer that learns how to weight mechanistic and observational signals under uncertainty (34).

The second decisive change is the shift from static risk prediction to patient-specific trajectories. Instead of generating a single risk score at a single time point, the twin should predict individualized growth curves, estimate the probability of growth acceleration, and provide projections of time to reach the threshold under uncertainty. These longitudinal predictions are more aligned with clinical decision-making than binary risk classification because they can directly inform surveillance intensity and intervention timing (12, 14).

In practice, each follow-up scan and biomarker panel should update the twin's status by narrowing the uncertainty when observed behavior matches predicted trajectories and by widening or shifting the uncertainty when the patient deviates from expected remodeling patterns.

Interpretability and confidence are essential because these outputs influence high-risk decisions. Explainability methods should be used to communicate which factors drive risk in a particular patient and how the contributions are distributed across domains (e.g., whether risk is primarily driven by rapid prior growth, localized peak wall stress, impaired hemodynamics, or persistent inflammation). The cardiovascular AI literature has highlighted the need for transparent decision support and careful avoidance of misleading correlations (10). Multimodal data fusion integrating imaging, hemodynamic and inflammatory biomarkers, and increasingly multiple omics layers, enables course prediction and personalized risk modeling (35).

Equally important, predictions should be presented with explicit uncertainty limits (e.g., prediction ranges for future diameter/volume, confidence intervals for growth rate) rather than single deterministic outputs. Uncertainty-aware reporting is critical to avoiding false sensitivity, especially when modeling assumptions (boundary conditions, wall features, biomarker variability) significantly impact predictions.

A defining characteristic of a living digital twin is adaptive learning. The prediction layer should incorporate real-world outcomes such as observed growth rates, post-EVAR/TEVAR sac regression, and complications into the recalibration of both mechanistic parameters and data-driven fusion weights. Contemporary digital twin reviews highlight that sustainable clinical translation relies on performance monitoring, deviation detection, and model updating mechanisms rather than a one-off build-and-deploy approach (12, 14).

At scale, registry-linked learning cycles and multi-center updates can strengthen generalizability, while federation strategies can help address data management and privacy constraints in distributed healthcare settings (13).

Finally, since Domain 4 functions as a clinical decision support system, it must be designed with translation constraints in mind. Digital twin publications in the field of cardiovascular medicine highlight the importance of reproducible pipelines, careful dataset documentation, subgroup performance evaluation, bias monitoring, and post-deployment oversight; these principles are consistent with regulatory expectations for AI-powered software used in clinical care (13, 14).

Establishing these principles early strengthens the clinical reliability of the twin and provides a clear path toward implementation.

#### 2.4.1 Digital twin implication

Domain 4 provides the “cognitive layer” of the aortic digital twin, integrating multimodal signals from Domains 1–3 into interpretable and uncertainty-aware predictions that evolve as longitudinal data accumulates. Without this layer, the previous domains remain descriptive. With this layer, the digital twin becomes a trajectory-oriented decision support system capable of informing precision endovascular strategy, along with individualized monitoring, risk stratification, and intervention modules.

### 2.5 Domain 5: precision endovascular strategy and virtual intervention planning

While preceding domains describe and interpret the evolving state of aortic disease, Domain 5 translates these insights into a personalized procedural strategy. At this stage, the digital twin moves from trajectory prediction to intervention simulation, enabling patient-specific endovascular repair and post-operative monitoring planning (36).

Traditional endovascular planning relies primarily on geometric measurements, assessment of landing zones, evaluation of angles and curves, and adherence to device usage instructions. While this approach has significantly improved outcomes, it does not systematically incorporate patient-specific hemodynamics, stress distribution after graft placement, or the potential for biological remodeling of the aneurysm wall. AI-based models can enable more personalized endovascular planning by assisting with device sizing, landing site assessment, and complication risk prediction (37). A digital twin-supported strategy integrates these domain frameworks into a unified decision support framework, aligning structural mechanics, flow dynamics, and immune status with procedural planning (12, 14).

In addition, computational constraints remain a practical limitation. High-accuracy simulations, such as fluid-structure interaction (FSI) modeling, can require significant computational time, limiting real-time applicability in acute or intraoperative situations. Therefore, simplified or surrogate modeling approaches may be necessary for time-sensitive decision-making.

#### 2.5.1 Virtual device placement and sealing integrity

Patient-specific digital twins allow for the simulation of stent-graft placement within reconstructed anatomy using finite element and fluid-structure interaction approaches. This modeling enables the assessment of device-wall interaction, radial force distribution in proximal and distal descent zones, and predicted fit in anatomically challenging regions such as angled or short necks (15).

Instead of simply verifying geometric fit, virtual placement can assess how stress fields redistribute after graft placement, whether focal stress concentrations persist, and how flow patterns change in the excluded sac and adjacent branching vessels.

Importantly, the integration of Domains 1 and 2 allows for the simulation of both mechanical stabilization and hemodynamic normalization. This integrated assessment goes beyond binary determinations of “device fits” to a quantitative assessment of whether the intervention significantly reduces wall stress and adverse flow environments.

##### 2.5.1.1 Digital twin implication

Virtual placement should measure not only anatomical compatibility but also post-operative stress distribution and flow modification.

#### 2.5.2 Endoleak risk and sac evolution modeling

Endoleak is a central predictor of long-term EVAR and TEVAR outcomes. Traditional follow-up strategies typically identify leaks retrospectively after sac expansion has occurred. In contrast, digital twin can simulate potential hemodynamic pathways leading to persistent sac pressurization.

Computational hemodynamics can identify regions prone to flow recirculation, residual pressure transmission, or backfilling from branched vessels, which increase susceptibility to type II endoleak (5).

Biomechanical matching further enables assessment of whether sac wall stress is adequately reduced after simulated exclusion or remains high in specific regions despite technically successful graft placement.

When immunobiological data are included, the twin can account for persistent inflammatory activation that may impair sac regression independently of overt hemodynamic leakage. This integrative approach is particularly important given the well-known heterogeneity of sac remodeling after repair. Digital twin inference: post-intervention modeling should predict sac volume trajectories under uncertainty, rather than relying solely on categorical leakage detection.

#### 2.5.3 Device strategy optimization

In complex anatomy, device selection often requires balancing compatibility, radial strength, branching preservation, and long-term durability. Digital twin-based planning enables comparative simulation of multiple device configurations within the same patient-specific anatomy.

Such simulations can assess sealing zone integrity, stress concentration at fixation points, predicted migration risk under vibrational loading, and branch vessel flow preservation (38). By combining mechanical simulation with predictive intelligence (Domain 4), the twin can predict not only the immediate mechanical feasibility but also the predicted long-term remodeling behavior under each device scenario.

This scenario-based optimization is consistent with broader cardiovascular digital twin frameworks that emphasize intervention simulation as a fundamental translational application (13, 14).

Instead of selecting a device based solely on geometric criteria, clinicians can ultimately compare simulated outcomes across strategies and choose the configuration that most favorably alters the predicted disease course.

##### 2.5.3.1 Digital twin implication

Device selection becomes a trajectory optimization problem rather than a purely geometric matching exercise.

#### 2.5.4 Post-repair surveillance personalization

After endovascular repair, surveillance protocols are often standardized according to time-based schedules rather than individualized risk. Digital twin allows for dynamic adjustment of follow-up intensity based on predicted sac regression, residual biomechanical stress distribution, hemodynamic normalization, and evolving immune activity.

Patients showing stable stress reduction, normalized flow fields, and decreased inflammatory signals may require longer imaging intervals, while persistent stress concentrations or inflammatory activation may justify intensified follow-up. In this way, surveillance shifts from uniform scheduling to risk-adaptive monitoring guided by integrated mechanistic and predictive modeling.

##### 2.5.4.1 Digital twin implication

Surveillance strategies should reflect individualized trajectory predictions and uncertainty estimates rather than fixed temporal algorithms.

#### 2.5.5 Translational considerations for clinical integration

For digital twin-guided intervention planning to become clinically feasible, computational modeling must be compatible with procedure timelines and workflow constraints. Simulation turnaround times should be consistent with preoperative planning, and outputs should be presented through intuitive interfaces that communicate stress distribution, predicted sac development, and uncertainty in a clinically interpretable manner.

As highlighted in the contemporary cardiovascular digital twin literature, successful implementation is more dependent on reproducibility, transparency, and integration into existing care pathways than on maximum computational complexity (12, 14).

Prospective evaluation will determine whether the digital twin-guided strategy improves complication rates, reduces re-intervention, and optimizes resource utilization.

## 3 Clinical validation, implementation, and evidence roadmap

The conceptual architecture of a patient-specific aortic digital twin is only as meaningful as its clinical validation and implementation pathway. While Domains 1–5 describe a mechanistically integrated system capable of modeling geometry, hemodynamics, immunobiology, predictive intelligence, and intervention strategy, translating it into routine care requires a systematic assessment across technical, clinical, regulatory, and economic dimensions. Current digital twin literature in cardiovascular medicine emphasizes that the critical hurdle is not computational feasibility, but reproducibility, workflow integration, and demonstrable clinical impact (12, 14, 38).

### 3.1 Technical validation and model calibration

Before clinical adoption, each component of the twin must undergo internal and external validation. Structural reconstructions require reproducible segmentation and geometric consistency across serial imaging. Hemodynamic simulations must demonstrate stability under boundary condition variation and include uncertainty quantification. Biomechanical outputs should be compared with established rupture risk studies (6, 19); immunobiological integration should demonstrate a biologically plausible match rather than statistical overfitting.

Importantly, validation should extend beyond component accuracy to system-level consistency. For example, when longitudinal imaging shows faster-than-predicted growth, recalibration of remodeling parameters should improve subsequent predictions. This iterative calibration process transforms the twin from a static model into an adaptable clinical tool.

### 3.2 Retrospective and prospective clinical evaluation

A pragmatic validation pathway might begin with retrospective cohort testing where the digital twin is built from historical imaging and biomarker datasets to assess the prediction of growth, rupture, or post-intervention outcomes. Performance metrics should include discrimination, calibration, and uncertainty assessment across clinically relevant subgroups (e.g., infrarenal AAA, thoracic aneurysm, dissection). However, retrospective validation alone is insufficient. Prospective evaluation is essential to determine whether digital twin-guided decision support alters clinical behavior and improves outcomes. A phased approach might include:

- Feasibility studies evaluating build time and integration into pre-procedural workflows
- Blind prediction trials comparing predicted and observed trajectories
- Intervention trials evaluating whether twin-guided timing or device selection reduces complications or re-intervention

This type of phased validation is consistent with broader cardiovascular AI application frameworks that emphasize prospective impact assessment rather than just retrospective performance claims (13, 14).

### 3.3 Workflow integration and clinical usability

Even highly accurate models fail if they disrupt the workflow. For aortic digital twins to become usable, the model building process needs to be accomplished within clinically acceptable timeframes, ideally integrated into radiology and vascular surgery planning processes. Outputs should be presented through intuitive dashboards displaying the following information:

- Predicted growth trajectories with uncertainty bands
- Spatial overlaps of stress and inflammation
- Scenario comparisons for device strategies

Successful clinical implementation requires standardized data acquisition protocols and data harmonization. For example, 4-dimensional flow MR parameters such as spatial resolution, temporal resolution, and velocity encoding (VENC) settings need to be defined consistently to ensure reproducibility. In many real-world clinical settings, such data may be incomplete or inconsistently recorded, representing a significant obstacle to the immediate deployment of comprehensive digital twin models. A pragmatic implementation pathway may define minimum data requirements, including baseline CTA imaging, basic clinical covariates, and longitudinal diameter measurements, with optional integration of advanced hemodynamic and immunobiological data as available. In practice, inconsistent recording of sequence parameters, contrast timing, and acquisition metadata may further limit reproducibility across centers.

Importantly, explainability tools (Domain 4) should be embedded in these interfaces to support clinician interpretation and collaborative decision-making. Experiences from cardiovascular AI show that adoption largely depends on transparency, ease of interpretation, and minimal additional cognitive load (10).

### 3.4 Regulatory and ethical considerations

Since digital twins function as clinical decision support systems, regulatory pathways such as FDA Software as a Medical Device (SaMD) frameworks and emerging European AI governance mechanisms are of direct importance (39). Cardiovascular digital twin analyses highlight the need for clearly defined training datasets, predefined update protocols, bias assessment among demographic subgroups, and post-deployment surveillance plans (13, 14).

Adaptive learning introduces additional complexity: models updated over time require predefined change management strategies to ensure that performance improvements do not compromise safety or create unwanted bias. Therefore, transparent documentation of the update logic and real-world monitoring are key components of the evidence roadmap. Model validation should include retrospective benchmarking, prospective observational validation, and post-implementation performance monitoring. Mitigations against unstable or unreliable predictions may include uncertainty thresholds, outlier detection, and validation systems under clinical expert supervision to ensure predictions remain interpretable and clinically relevant.

Depending on the task, prediction components may include mechanistic simulation models, supervised machine learning classifiers, longitudinal prediction models, and hybrid physics-informed machine learning systems.

### 3.5 Health economic assessment and value proposition

Large-scale implementation requires a demonstration of value. Digital twin construction incurs costs associated with image processing, computational resources, biomarker profiling, and expert interpretation. These should be balanced with potential benefits, including:

- Avoiding unnecessary early intervention
- Preventing rupture and emergency surgery
- Reducing re-intervention rates
- Optimizing surveillance intervals

Cost-effectiveness analyses, including quality-adjusted life years (QALY) and health system expenditures, will be critical to justifying reimbursement pathways and widespread adoption. Importantly, the economic assessment should account not only for upfront costs but also for subsequent savings resulting from reduced complications.

### 3.6 Towards continuously learning healthcare systems

In conclusion, the full promise of aortic digital twins lies in their integration into learning healthcare systems. Record-linked data, multi-center collaborations, and unified learning architectures can support continuous recalibration and cross-population validation while protecting data privacy. Such infrastructures allow the twin to evolve as device technology, medical treatment, and population characteristics change. This continuous learning paradigm is consistent with recent digital twin visions in cardiovascular medicine, where mechanical simulation and data-driven inference coexist within adaptive ecosystems (12, 14).

## 4 Future horizons: towards therapeutic and networked digital twins

The framework outlined above positions patient-specific aortic digital twins as integral decision support systems capable of predicting disease course and informing precise intervention. However, the next stage of development extends beyond prediction and procedural planning to therapeutic simulation, multi-region cardiovascular integration, and continuously learning ecosystems that are updated as new patient data, devices, and treatments emerge. These trends are already anticipated in the broader cardiovascular digital twin literature, where application scale challenges, multimodal fusion, and clinical translation are increasingly emphasized (12, 14).

### 4.1 From predictive twins to therapeutic twins

To date, most cardiovascular digital twin applications—especially those proposed for vascular diseases—have been framed primarily as tools for risk estimation, surveillance planning, and virtual procedural testing. A logical next step is the therapeutic digital twin, which explicitly models how medical treatment affects biological activity, mechanistic loading, and remodeling kinetics. This evolution is particularly important in aortic aneurysm disease, where progression reflects conjugated biomechanical processes and can potentially be modified by therapies that alter inflammation, proteolysis, extracellular matrix turnover, or hemodynamic load (8).

Operationally, therapeutic simulation requires robust mappings between measurable biological signals (e.g., inflammatory biomarkers, immune cell phenotypes, or imaging-based measures of inflammation) and model parameters representing remodeling susceptibility or wall weakening. Even if such mappings remain probabilistic rather than deterministic, incorporating treatment response uncertainty into the digital twin can enable individualized predictions of expected benefit and guide “risk mitigation” strategies in a measurable way. In this paradigm, the clinical focus expands beyond the binary decision of whether or not to perform surgery, towards identifying the optimal combination of medical and procedural strategies that can most positively alter an individual patient's predicted course.

### 4.2 Integration in cardiovascular territories

Aortic disease frequently occurs in conjunction with coronary, carotid, and peripheral artery pathology, and many factors contributing to vascular fragility are systemic. Furthermore, ventricular-arterial coupling shapes aortic loading conditions, while systemic blood pressure control and arterial stiffness influence biomechanical stress distributions. These facts motivate future digital twin architectures that move beyond single-zone modeling and toward networked cardiovascular twins, integrating cardiac output dynamics, systemic vascular impedance, and multi-bed atherosclerotic load into a unified, physiology-sensitive representation (12, 14).

Such integration will allow for more direct address of clinically meaningful questions: how changes in ventricular function, blood pressure targets, or systemic inflammation alter aortic stress and remodeling; and conversely, how aortic pathology affects afterload and downstream perfusion. Importantly, multi-zone integration does not require maximum complexity for each patient; rather, it supports modular applications where the depth of modeling is adjusted according to clinical needs and data availability.

### 4.3 Foundation models and transfer learning in vascular diseases

As aortic and cardiovascular datasets expand, digital twin systems can increasingly incorporate underlying model paradigms, including pre-trained multimodal models that provide adaptable baseline parameterizations to individual patients through fine-tuning. This approach can mitigate the “cold start” problem of patient-specific calibration and accelerate deployment in settings where comprehensive local datasets are unavailable. Transfer learning can be extremely valuable, particularly in rare aortic phenotypes (e.g., genetic syndromes, atypical dissection variants) where single-center sample sizes are inherently limited.

At the same time, scale-oriented learning must be balanced with the need for mechanistic accuracy. Hybrid architectures that leverage deep representations for image-derived features, temporal trends, or multimodal fusion while maintaining explicit biomechanical connectivity can offer a pragmatic middle ground that improves generalizability and calibration while preserving interpretability and causal structure (13).

### 4.4 Regenerative and bioengineering applications

Emerging regenerative strategies, including gene-based extracellular matrix synthesis modulation, biomaterial-driven vascular repair, and tissue-engineered grafts, are introducing novel variables in aortic disease management that are difficult to assess alone with conventional imaging methods. A mature aortic digital twin can serve as an in-silico testing environment to investigate the predicted impact of collagen/elastin turnover-altering therapies, model host-material integration dynamics, and predict local immune responses that may affect healing and long-term stability.

While these applications are still in the exploratory phase, their conceptual value is immediately apparent: by embedding regenerative and bioengineering interventions within a digital twin framework, novel therapies can be stress-tested in a computer environment before widespread clinical adoption, thereby strengthening translational safety and hypothesis generation.

### 4.5 Ethics, equity and global considerations

As digital twin outputs increasingly influence surveillance intervals, intervention timing, and device selection, equity and governance are becoming central concerns rather than peripheral ones. Ensuring consistent performance across gender, age, ancestry, and socioeconomic strata is essential to prevent the exacerbation of existing inequalities. Practical measures include structured supervision, bias monitoring, transparent reporting of subgroup performance, and the deliberate inclusion of underrepresented populations during model development (12, 14).

Global scalability also requires explicit planning. Many centers will lack access to advanced computational hemodynamic pipelines or molecular imaging. Accordingly, incremental or modular architectures where the twin can operate using imaging and clinical data, and incrementally integrate hemodynamics and immunobiology as available, can enhance accessibility and applicability in healthcare settings while maintaining conceptual integrity.

### 4.6 Long-term vision: a living cardiovascular ecosystem

Ultimately, patient-specific digital twins should be viewed not as isolated software tools, but as nodes within a learning cardiovascular ecosystem. Serial imaging, laboratory profiles, wearable device signals, and longitudinal outcome logging can support continuous recalibration, deviation tracking, and outcome-related improvement. As new devices, materials, and treatments emerge, their effects can be incorporated into prediction frameworks and tested on virtual cohorts, accelerating evidence generation while maintaining patient-specific relevance.

This trend parallels a broader shift in cardiovascular medicine: from static, threshold-based decision rules to dynamic, individualized risk trajectories supported by continuous data streams and adaptive modeling. In this paradigm, the aortic digital twin functions both as a disease-specific application and as a prototype for system-level integration across cardiovascular domains (12, 14).

## 5 Conclusion

Patient-specific digital twins offer a structured pathway to integrate geometry, mechanics, hemodynamics, and immunobiology into individualized trajectory-based modeling of aortic disease. By focusing on dynamic, patient-specific evolution from static anatomical thresholds, this framework has the potential to improve risk stratification, guide precise intervention, and personalize monitoring strategies. Realizing this vision will require careful validation, responsible implementation, and sustainable interdisciplinary collaboration.
